# 1321. CT Brain Prior to Lumbar Puncture in Patients with Suspected Meningitis: A Single Centre Study of Compliance with Expert Guidelines and Diagnostic Yield of Imaging.

**DOI:** 10.1093/ofid/ofad500.1160

**Published:** 2023-11-27

**Authors:** Philip J Dempsey, Fergus O’Herlihy, Brian Thomas Gibney

**Affiliations:** Mater Misericordiae University Hospital, Dublin, Dublin, Ireland; Mater Misericordiae University Hospital, Dublin, Dublin, Ireland; Mater Misericordiae University Hospital, Dublin, Dublin, Ireland

## Abstract

**Background:**

Neuroimaging prior to lumbar puncture (LP) in suspected meningitis was once thought necessary to outrule raised intracranial pressure (ICP) which would risk brainstem herniation. However, the frequency of a lesion causing raised ICP is low and the risk of herniation post LP is minimal. The inappropriate use of neuroimaging prior to LP can delay diagnosis and reduce the diagnostic yield of CSF culture results. Several expert groups have published guidelines to reduce unnecessary neuroimaging in these patients. The purpose of this study was to review the use of CT brain prior to LP in our institution to identify which guidelines can be safely followed and the diagnostic yield of neuroimaging.

**Table 1:**
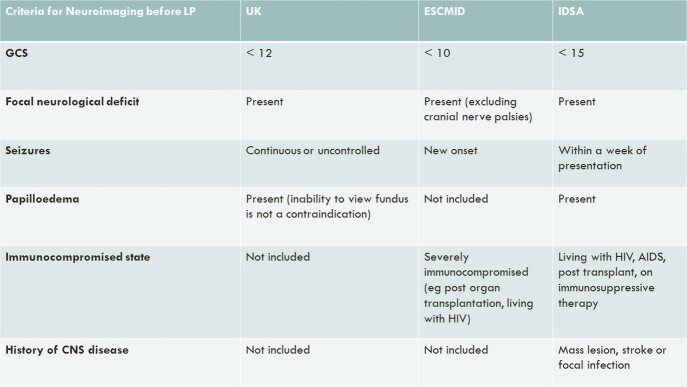
Criteria for CT Brain before Lumbar Puncture

The recommendations for when neuroimaging is indicated before lumbar puncture in suspected meningitis as per the UK, European Society of Clinical Microbiology and Infectious Diseases and Infectious Diseases Society of America guidelines.

**Methods:**

The picture archiving and communications system was retrospectively reviewed from March 2013 to March 2023 to identify patients who had been referred for CT brain to facilitate LP for suspected meningitis. The referral information was reviewed and compared to the UK, Infectious Disease Society of America and European guidelines. The imaging findings were recorded to assess for any abnormality and the patient EMR was reviewed to assess for any clinical deterioration post LP.

**Results:**

196 patients were included. The mean time from presentation to CT was 6.5 hours. 19, 28 and 65 of the 196 studies were indicated as per the UK, EU and IDSA guidelines respectively, resulting in a 90%, 85% and 67% reduction in imaging if these guidelines were followed. Of the 177 studies deemed inappropriately performed by the UK guidelines, 164 (93%) were normal. 12 (7%) had findings that did not preclude LP. One patient (< 1%) had a perimesencephalic haemorrhage. 19 (10%) studies were indicated, 16 of which were normal (84%). Of the remaining 3 (2%), none of the CT findings precluded LP. The application of the IDSA and European guidelines did detect any additional findings that precluded LP.

**Conclusion:**

The use of expert guidelines is an important tool in reducing unnecessary neuroimaging and delays to lumbar puncture and treatment for patients with suspected meningitis. This study supports the use of the UK guidelines over other guidelines, which resulted in higher numbers of patients imaged without any additional clinical benefit. Red flag symptoms for intracranial haemorrhage should be carefully screened for.

**Disclosures:**

**All Authors**: No reported disclosures

